# Coping with the COVID-19 pandemic – the role of leadership in the Arab ethnic minority in Israel

**DOI:** 10.1186/s12939-020-01257-6

**Published:** 2020-09-09

**Authors:** Mor Saban, Vicki Myers, Rachel Wilf-Miron

**Affiliations:** 1 Gertner Institute for Health Policy and Epidemiology, Ramat Gan, Israel; 2 Tel- Aviv univesrity, School of Public Health, Sackler Faculty of Medicine, Ramat Gan, Israel

**Keywords:** Arab, Ethnic, COVID-19, Israel, Leadership, Ultra-orthodox Jew

## Abstract

**Background:**

The Arab ethnic minority makes up 21% of Israel’s population, yet comprised just 8.8% of confirmed cases and 3.6% of deaths from COVID-19, despite their higher risk profile and greater burden of underlying illness. This paper presents differences in patterns of morbidity and mortality from COVID-19 in the Arab, ultra-Orthodox and overall populations in Israel, and suggests possible reasons for the low rates of infection in the Arab population.

**Methods:**

Data were obtained from the Israeli Ministry of Health’s (MOH) open COVID-19 database, which includes information on 1270 localities and is updated daily. The database contains the number of COVID-19 diagnostic tests performed, the number of confirmed cases and deaths in Israel.

**Results:**

In the first 4 months of Israel’s COVID-19 outbreak, just 2060 cases were confirmed in the Arab population, comprising 8.8% of the 23,345 confirmed cases, or 2.38 times less than would be expected relative to the population size. In contrast, the ultra-Orthodox made up 30.1% of confirmed cases yet just 10.1% of the population. Confirmed case rate per 100,000 was twice as high in the general Jewish population compared to the Arab population. The Arab mortality rate was 0.57 per 100,000, compared to 3.37 in the overall population, and to 7.26 in the ultra-Orthodox community. We discuss possible reasons for this low morbidity and mortality including less use of nursing homes, and effective leadership which led to early closure of mosques and high adherence to social distancing measures, even during the month of Ramadan.

**Conclusions:**

Despite a disproportionate burden of underlying illness, the Arab population did not fulfil initial predictions during the first wave of the COVID-19 outbreak and maintained low numbers of infections and deaths. This contrasts with reports of increased mortality in ethnic minorities and economically disadvantaged populations in other countries, and with high rates of infection in the ultra-Orthodox sector in Israel. Effective leadership and cooperation between individuals and institutions, particularly engagement of community and religious leaders, can reduce a group’s vulnerability and build resilience in an emergency situation such as the current pandemic.

## Introduction

The COVID-19 pandemic has affected, at the time of writing (1/7/20) over 200 countries, with 10,495,019 confirmed cases, and 511,686 deaths. Various countries have reported social disparities, with racial and ethnic minority groups being hit harder, suffering disproportionately greater infection and mortality rates [1]. COVID-19 mortality was markedly higher for black Americans (61.6 deaths per 100,000) compared to all other ethnic groups (white Americans 26.2 deaths per 100,000; Latino Americans 28.2 deaths per 100,000) [2]. In Wales (UK) mortality was twice as high in the most deprived compared to the least deprived areas [3]. Among healthcare workers in the UK, two thirds of fatalities from COVID-19 between February and April were in black or ethnic minority workers [4]. Public Health England published a report into the disproportionate number of cases experienced by ethnic minorities, reporting that people from ethnic minorities had between 10 and 50% higher risk of death from COVID-19 compared to white British [5]. The report suggested that the added risk was both in likelihood of exposure (through occupation, housing density, use of public transport), and in complications and mortality once infected (due to more pre-existing health conditions like diabetes).

In Israel, 23,345 confirmed cases and 308 deaths were reported between 21st February and 25th June 2020. Between March 31st and May 1st, the rate of infection was 2.16 times higher in the lowest compared to the highest socioeconomic groups [6]. Minority groups in Israel considered at the outset to be at greater risk due to lower socioeconomic status and higher incidence of risk factors, included the Arab population and the ultra-Orthodox Jews.

The Arab population comprises 21.0% of a total 9.1 million population of Israel and the ultra-Orthodox community a further 10.1% of the population. Household income and participation in the workforce is lower in both these groups compared to the general Jewish population. Housing is more crowded with 26.5% of Arab families having more than 2 people per room, compared to 4.6% in the general Jewish population, but similar to the ultra-Orthodox (26%) [7], although living density in ultra-Orthodox neighbourhoods (which are more central and city-based) is generally higher, while housing in Arab villages (generally more rural) is more spaced out. Overcrowded housing was reported to be related to increased mortality from covid-19 in a hospital based cohort [8].

This paper presents differences in patterns of morbidity and mortality from COVID-19 in the Arab, ultra-Orthodox and overall populations in Israel, and suggests possible reasons for the low rates of infection and mortality in the Arab population.

## Methods

Data were obtained from the Israeli Ministry of Health’s (MOH) open COVID-19 database from February 27st (the patient zero case) to June 25th, 2020 (4 consecutive months) (https://datadashboard.health.gov.il/COVID-19/?utm_source=go.gov.il&utm_medium=referral, and Telegram application- https://t.me/MOHreport), which includes information on 1270 localities and is updated daily. The database contains the number of COVID-19 diagnostic tests performed, the number of confirmed cases (i.e., those that tested positive by real-time quantitative reverse-transcriptase polymerase-chain-reaction [qRT-PCR] assay), the number of recovered cases, and deaths in Israel. Data on tests performed, confirmed COVID-19 cases and deaths were analyzed by population group (Arab, ultra-Orthodox Jewish, non-Orthodox Jewish). Population size was obtained from the Central Bureau of Statistics’ database [9].

## Results

In the first 4 months of Israel’s COVID-19 outbreak, just 2060 cases were confirmed in the Arab population, comprising 8.82% of the 23,345 confirmed cases, or 2.38 times less than would be expected relative to the population size (Table 1) [10]. In contrast, the ultra-Orthodox made up 30.08% of confirmed cases yet just 10.1% of the population. Confirmed case rate per 100,000 was twice as high in the general Jewish population compared to the Arab population, and three times higher in the ultra-Orthodox population compared to the overall population rate.

**Table 1:** Confirmed COVID-19 cases, deaths and testing rates from February to June 2020 by population group

	Population	Cases	Deaths	Tests	Case rate/ 100,000	Death rate/ 100,000	Testing rate/ 1000
**Arabs**	1,916,000	2060	11	76,532	107.5	0.57	39.9
**Ultra-Orthodox**	922,736	7023	67	81,961	761.1	7.26	88.8
**Non-Orthodox Jews**	6,297,264	14,262	230	691,350	226.5	3.65	109.8
**Total Population**	9,136,000	23,345	308	849,849	255.5	3.37	93.0

**Fig. 1:**
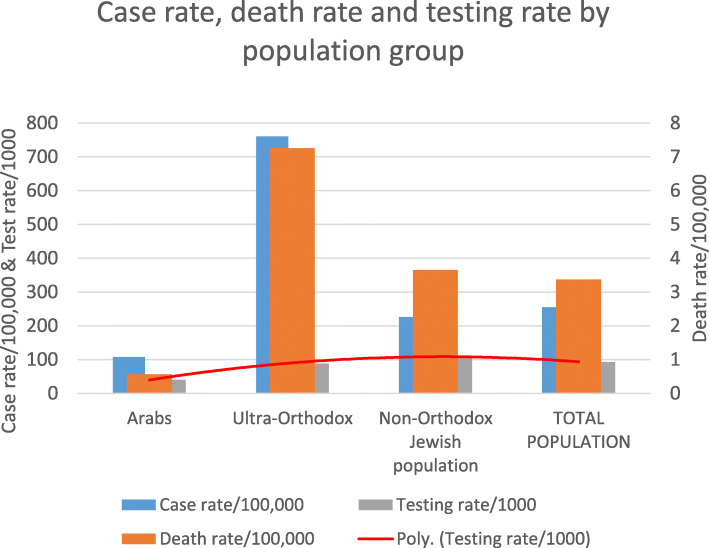
Confirmed case rate, mortality rate and testing rate by population group

Of 308 deaths, only 11 were identified as from the Arab population (3.6%), 5.8 times less than expected for their population size. The Arab mortality rate was 0.57 per 100,000, compared to 3.37 in the overall population, and to 7.26 in the ultra-Orthodox community (Fig. 1).

Testing rates were lower in the Arab population (Fig. 1), with 39.9 per 1000 compared to 93 per 1000 in the general population, potentially identifying fewer cases, however this does not explain the significantly lower mortality rate. The proportion of positive tests was 2.74% overall, 2.69% in the Arab population, and 8.57% in the ultra Orthodox population.

## Discussion

During the first wave of the COVID-19 pandemic in Israel, two population minority groups were predicted to be at high-risk. The ultra-Orthodox ‘fulfilled’ projections with a rate of infection almost 3 times higher than expected for the population segment [10]. Waitzberg and her colleagues described the unique cultural and behavioral norms of this sector, which contributed to accelerated viral spread [11].Among those norms are the tendency among some ultra-Orthodox communities to follow the instructions of their own religious leadership which do not necessarily coincide with governmental recommendations, and the intense social and community life, both resulting in the lack of physical distancing. In contrast, in the Arab population, characterized by similarly low socioeconomic status as the ultra-Orthodox, infection and mortality rates were very low. These lower rates seem to contrast with findings in other countries where ethnic minorities are at greater risk.

These data are surprising not only due to the ethnic minority status and weaker socioeconomic position of the Arab population, but also due to higher risk profile among this sector, with higher incidence of risk factors and chronic illness known to increase risk of mortality. For example diabetics have a 9-fold increased risk of serious illness from COVID − 19 compared to non-diabetics [12], yet diabetes is 1.79 times more common in the Arab compared to the Jewish population (controlled for age and sex) in Israel [13].

We suggest several explanations that may account for the surprisingly low morbidity and mortality from Covid-19 in the Israeli Arab population:

1. **Young population:** Age is the most significant risk factor for serious illness and complications from COVID -19 [12]. The Arab population is generally younger than the Jewish population (mean age 26 vs. 34), with 4% aged over 65 [14], though this is also true of the ultra-Orthodox sector, with just 3% aged over 65, compared to 15% in the overall Jewish population [15, 16].
2. **Fewer nursing homes:** 45% of mortality from the virus in Israel was reported in nursing homes [17]. In Arab society, it is traditional for families to care for older members at home, and less common to use outside care services [18]. Care homes are mostly used for short stays for rehabilitation and medical care [19]. Nursing homes are also far less available in Arab, compared with Jewish, localities. In comparison, in the ultra-Orthodox community, care homes are used more widely. Of the 148 care homes registered with the Ministry of Labor, Social Affairs and Social Services, just 4 (2%) are in Arab towns, while there are 17 in Jerusalem (36% ultra-Orthodox population) and 5 in Bnei Brak (an ultra-Orthodox town), making up 15% of institutions [20]. Indeed confirmed cases and deaths did occur in designated ultra-Orthodox care homes.
3. **Cooperation between community leaders, local authorities and governmental bodies**: The Israeli Arab population comprises a strong community with a sense of solidarity [21]. This social cohesion facilitated internal organization during the COVID-19 crisis. An unprecedented collaboration arose between the Ministries of Health, Welfare, Internal Affairs and Homeland Security and the Arab community, including local authorities, civil society, healthcare professionals and opinion leaders. A dedicated “situation room” coordinated between the Ministries and the community and helped convey the unique needs and requests from the field to policy makers. For example, early on it was clear that sub-optimal testing might interfere with understanding the infection status within the Arab community; a dedicated Arab call center was organized within the Israeli emergency medical services, to allow people to report potential symptoms or exposure to known cases and request testing. Instructions regarding the importance of social distancing, especially during Ramadan, were culturally tailored, recruiting religious leaders, physicians, politicians and Arab football stars to convey an effective message to relevant audiences. In contrast, the religious leaders of the ultra-Orthodox community did not heed early warnings and kept religious and educational establishments open, despite governmental instructions.
4. **Religious and political leaders promoted social distancing:** Cooperation between Muslim, Christian and Druze religious leaders presenting a united front led to the closure of all places of worship, which were understood to be a key route of transmission. Since 85% of Israeli Arabs are Muslim [15], the actions of religious leaders (Muftis) played an important role. Among other steps, a formal religious order (fatwa) was issued to follow the Ministry of Health instructions and local authority rules to conduct small funerals, and to avoid large gatherings. One of the most significant steps was the early closure of mosques (17/3/20), before synagogues were closed. This step was critical in preventing infections particularly during Ramadan, a time when families traditionally gather in prayer and celebration. The decision to close the Al-Aqsa mosque in Jerusalem during Ramadan was of great importance and religious significance in saving lives. The closure of mosques was fully adhered to, without attempts to open alternative places of worship, as happened among the ultra-Orthodox Jews. In parallel, the Arab political leadership, which won increased support in the last elections, also called for Arab citizens to cooperate with the preventive measures, and their message had an impact.
5. **Lower rate of travel abroad:** The COVID-19 outbreak in Israel was largely spread by travelers returning from abroad. The number of people travelling abroad is lower in the Arab population compared with the general Israeli population. Several hundreds of ultra-Orthodox Jews, a sector that usually travels less than the general Jewish population (17% vs 52% in 2017–18) [22], returned from abroad during the outbreak, some from communities with high infection rates and indeed many of them tested positive.
6. **High representation of Arabs in medical professions:** There is a high rate of Arab doctors, nurses and pharmacists, compared to those in the ultra-Orthodox population, which is under-represented in medicine and nursing [23]. Arabs make up 18.4% of nurses, yet just 12.8% of the general workforce [24]. Medical teams, although at higher risk of infection through exposure at work, contributed to dissemination of messages in their communities regarding the reasons for, and importance of, following guidelines, and how to maintain hygiene and social distancing. This was particularly illustrated by the head of emergency medicine at a major hospital (Rabin Medical Center), Dr. Riad Majadla, who broadcast health messages in Arabic on social media and radio, concerning the importance of hygiene and social distancing to prevent spread of the virus, including live broadcasts from areas with outbreaks, and guidelines of the Islamic council, and continues to do so; and the director of Ziv Medical Center, Dr. Salman Zarka, of the Druze community, who posted similar messages on social media. These were private initiatives that reached a wide audience.
7. **Social media messages:** Social networks are accessed more among the Arab population compared to the ultra-Orthodox population, who spurn use of the internet. According to a survey conducted in 2017 by the Israeli Internet Society, social networks are used by 73 and 61% of the Jewish and Arab population, respectively; while 87 and 76%, respectively, used search engines to look for information on the internet [25].In contrast, only 33% of ultra-Orthodox Jews used the internet to look for information. This wider use of social networks in the Arab population allowed the broad dissemination of health messages.

While it has been suggested that countries with wider testing showed more confirmed cases, and although testing rates were lower in the Arab population, this does not explain the differences in mortality seen in the Arab population in Israel [26]. Furthermore the percentage of positive tests was much higher in the ultra-Orthodox population compared to the general population, and to the Arab population.

In light of multiple reports from around the world of greater risk of COVID-19 morbidity and mortality experienced by ethnic minorities and socioeconomically disadvantaged groups, it is important to understand the reasons behind lower infection rates in an ethnic minority group in a developed high-income country. Though the demographic makeup of Israel is very different from the US and UK, lessons can be learned. Similarities can be seen in the high burden of chronic illness found in the Arab population, and in the black and Asian ethnic minorities in the UK and US who have higher rates of obesity, cardiovascular disease and diabetes, less job security, and often live in more deprived and more peripheral areas. It is all the more surprising then that despite these disadvantages, and being overrepresented in the healthcare professions, the Arab community managed to avoid their predicted fate and curb the spread of the virus.

## Comparison to other countries

While Europe and North and South America have experienced high rates of COVID-19 morbidity and mortality, many countries in the Middle East have so far demonstrated less steep curves.

Lockdowns were imposed early on in both Jordan (9 deaths; mosques closed March 14th) and Lebanon (33 deaths; mosques closed March 15th), where schools and mosques were closed after the first few cases appeared, and strict border controls put in place [26]. In contrast Iran, which suffered a high burden of COVID-19-deaths (~ 11,000 deaths), did not close its mosques until a month after the first case was registered.

These countries share with the Israeli Arab population a young age demographic profile and lower use of nursing homes. A much lower proportion of deaths occurred in care homes for example in Jordan (9%) compared to Israel (45%) and worldwide (41%) [17]. In Israel 2% of the overall population reside in care homes – lower than many other European/Western countries [27].

In Norway, the rate of COVID-19 infections in Somali immigrants was much higher than that of the general population (1586 per 100,000 compared to 140 per 100,000). Beyond poorer and more crowded living conditions, a further explanation proposed was less access to mainstream media. To counter this, a campaign was targeted using YouTube for public health videos in Somali and a telephone hotline, contributing to numbers beginning to fall in recent weeks [28]. Similar efforts were made in the Israeli Arab population to distribute health message on social media, to design messages in Arabic in a culturally relevant manner, and recruit the support of religious and community leaders.

In summary, based on data from the study period in the first 4 months of the COVID-19 outbreak, the case of the Israeli Arab population testifies, in contrast to reports from other countries, that belonging to an ethnic minority or to an economically disadvantaged group does not always equate to poorer health outcomes. Despite a disproportionate burden of underlying illness, the Arab population did not fulfil initial predictions during the first wave of the COVID-19 outbreak and maintained low numbers of infections and deaths. Effective leadership and cooperation between individuals and institutions, particularly engagement of community and religious leaders, can reduce a group’s vulnerability and build resilience in an emergency situation such as the current pandemic. These strengths could be harnessed to address other health issues in ethnic minorities and specifically in the Arab population in Israel.

## Data Availability

All data available in the Ministry of health, Israel website- URL: https://govextra.gov.il/ministry-of-health/corona/corona-virus-en/

## Abbreviation

COVID-19: Corona Virus December 2019

## References

[1] Hooper M, Nápoles A, JAMA EP-S-, 2020 U. COVID-19 and Racial/Ethnic Disparities. jamanetwork.com [Internet] 2020 [cited 2020 May 24];Available from: https://jamanetwork.com/journals/jama/article-abstract/2766098. 10.1001/jama.2020.8598 PMC9310097 32391864

[2] APM Labs (2020). The color of coronavirus: COVID-19 deaths by race and ethnicity in the US.

[3] Office for National Statistics. Deaths involving COVID-19 by local area and socioeconomic deprivation: deaths occurring between 1 March and 17 April 2020: Off Natl Stat; 2020.

[4] Kirby T (2020). Evidence mounts on the disproportionate effect of COVID-19 on ethnic minorities. Lancet.

[5] Public Health England. Beyond the data: Understanding the impact of COVID-19 on BAME communities GOV.UK [Internet]. [cited 2020 Jul 1];Available from: https://www.gov.uk/government/publications/covid-19-understanding-the-impact-on-bame-communities.

[6] Saban, M. Sahchar, T. Miron, O. Wilf-Miron R. Effect of socioeconomic and ethnic characteristics on COVID-19 infection: The case of the Ultra-Orthodox and the Arab communities in Israel. medRxiv 2020;. 10.1007/s40615-021-00991-z PMC7938876 33686623

[7] Chernichovsky D, Bisharat B, Bowers L, SC BA (2017). The Health of the Arab Israeli Population. Jerusalem Taub Cent Soc Policy Stud Isr.

[8] Overcrowded Housing and COVID-19 Risk among Essential Workers - Public Policy Institute of California [Internet]. [cited 2020 Jul 1];Available from: https://www.ppic.org/blog/overcrowded-housing-and-covid-19-risk-among-essential-workers/.

[9] Central Bureau of Statistics. Israel localities [Internet]. 2020 [cited 2020 Jun 24];Available from: https://www.cbs.gov.il/he/publications/Pages/2019/יישובים-בישראל.aspx.

[10] Avi W (2020). A picture of the nation 2020.

[11] Waitzberg R, Davidovitch N, Leibner G, Penn N, Brammli-Greenberg S (2020). Israel’s response to the COVID-19 pandemic: tailoring measures for vulnerable cultural minority populations. Int J Equity Health.

[12] Caramelo F, Ferreira N, Oliveiros B. Estimation of risk factors for COVID-19 mortality - preliminary results. medRxiv. 2020 [cited 2020 May 22];Available from. 10.1101/2020.02.24.20027268.

[13] Jaffe A, Giveon S, Wulffhart L (2017). Adult Arabs have higher risk for diabetes mellitus than Jews in Israel. PLoS One.

[14] Shnoor YBS (2018). The 65+ population in Israel: statistical abstract 2018.

[15] TAUB CENTER For Social Policy Studies In Israel (2017). The singer series: state of the nation report 2017.

[16] Malach G, Cahaner L (2018). Statistical report on ultra-orthodox (Haredi) Society in Israel 2018.

[17] Comas-Herrera A, Zalakain J (2020). Mortality associated with COVID-19 outbreaks in care homes: early international evidence. Int LONG TERM CARE POLICY Netw.

[18] Weihl H (1995). Implementation of the Long-term care law in the Arab sector.

[19] Abdelmoneium AO, Alharahsheh ST (2016). Family home caregivers for old persons in the Arab region: perceived challenges and policy implications. Open J Soc Sci.

[20] Ministry of Labor, Social Affairs and Social Services [Internet]. [cited 2020 Jul 1];Available from: https://www.gov.il/en//departments/molsa.

[21] Jamal A. Arab minority nationalism in Israel: the politics of indigeneity: Taylor & Francis; 2011.

[22] Malach G & Cahaner L. Statistical Report on Ultra-Orthodox Society in Israel: Highlights - The Israel Democracy Institute [Internet]. 2019 [cited 2020 Jul 1]. Available from: https://en.idi.org.il/articles/29348.

[23] Sufer-Fridman H (2012). Integrating a minority group in the labor market: the case of the ultra-orthodox in Israel.

[24] Popper-Giveon A, Keshet Y, Liberman I (2015). Increasing gender and ethnic diversity in the health care workforce: the case of Arab male nurses in Israel. Nurs Outlook.

[25] Ganaim A (2018). The internet in Arab society in Israel - initial snapshot and policy recommendations.

[26] Mumtaz G. Providing context for COVID-19 numbers in the Arab region. Nat Middle East [Internet] 2020 [cited 2020 Jul 1];Available from: http://www.natureasia.com/en/nmiddleeast/article/10.1038/nmiddleeast.2020.45.

[27] Chernichovsky D, Kaplan A, Regev E, Stessman J (2017). Long-term Care in Israel: funding and organization issues. Taub Center, Jerusalem.

[28] Cookson, C. & Milne, R. Nations look into why coronavirus hits ethnic minorities so hard | Free to read | Financial Times [Internet]. [cited 2020 Jul 1];Available from: https://www.ft.com/content/5fd6ab18-be4a-48de-b887-8478a391dd72.

